# An Innovative Fusion-Based Scenario for Improving Land Crop Mapping Accuracy

**DOI:** 10.3390/s22197428

**Published:** 2022-09-30

**Authors:** Solmaz Fathololoumi, Mohammad Karimi Firozjaei, Asim Biswas

**Affiliations:** 1 School of Environmental Sciences, University of Guelph, Guelph, ON N1G 2W1, Canada; 2 Department of Remote Sensing and GIS, Faculty of Geography, University of Tehran, Tehran 1417853933, Iran

**Keywords:** land crop classification, voting, feature selection algorithms, uncertainty, satellite images

## Abstract

The accuracy of land crop maps obtained from satellite images depends on the type of feature selection algorithm and classifier. Each of these algorithms have different efficiency in different conditions; therefore, developing a suitable strategy for combining the capabilities of different algorithms in preparing a land crop map with higher accuracy can be very useful. The objective of this study was to develop a fusion-based framework for improving land crop mapping accuracy. First, the features were retrieved using the Sentinel 1, Sentinel 2, and Landsat-8 imagery. Then, training data and various feature selection algorithms including recursive feature elimination (RFE), random forest (RF), and Boruta were used for optimal feature selection. Various classifiers, including artificial neural network (ANN), support vector machine (SVM), and RF, were implemented to create maps of land crops relying on optimal features and training data. After that, in order to increase the result accuracy, maps of land crops derived from several scenarios were fused using a fusion-based voting strategy at the level of decision, and new maps of land crops and classification uncertainty maps were prepared. Subsequently, the performance of different scenarios was evaluated and compared. Among the feature selection algorithms, RF accuracy was higher than RFE and Boruta. Moreover, the efficiency of RF was higher than SVM and ANN. The overall accuracy of the voting scenario was higher than all other scenarios. The finding of this research demonstrated that combining the features’ capabilities extracted from sensors in different spectral ranges, different feature selection algorithms, and classifiers improved the land crop classification accuracy.

## 1. Introduction

Achieving sustainable food security is an important challenge. Population growth and declining agricultural land quality have led researchers to seek solutions to increase crop yield [1,2]. Moreover, water scarcity and environment-related issues are other factors that necessitate the development of new methods in agricultural management in order to increase yield in addition to optimizing inputs [3]. Therefore, the study of various aspects of precision agriculture has been a popular research topic in past few years [4]. Accurate identification of crop type and estimation of the crop area are essential for the implementation of a precise agricultural system and, ultimately, planning for food security [5].

Accurate identification of crop type and preparing land crop maps require access to appropriate field data. Collecting crop data comprehensively and accurately on a large scale is not possible using traditional methods [6]. According to previous studies, satellite data makes it possible to access this information in less time and at a lower cost. Remote sensing data—due to its appropriate and diverse spatial and radiometric resolution, and its multi-temporal, multi-spectral, wide, and integrated vision—is able to differentiate various agricultural phenomena including type, temporal, and spatial cultivation patterns. Furthermore, this technology can be used in various environmental domains, including monitoring and quantification of various agricultural land-related parameters, due to the frequency of image capturing from one area, and the development of algorithms for processing and rapid interpretation of the obtained data. Recent advances in sensors have further expanded the use of this technology in agricultural management [7,8].

Various studies have utilized satellite images for land crop classification. Kpienbaareh et al. [9] achieved accurate detailed crop type maps through combining Sentinel 1, Sentinel 2, and Planet Scope data using the RF algorithm. Landsat-8 and Sentinel 1 images were combined in the research by Inglada et al. [10], leading to an improvement in the classification of land crop results. Pott et al. [11] revealed that using Sentinel 1 and 2 images and Shuttle Radar Topographic Mission (SRTM) elevation models improved the overall accuracy of classification for land crops. The combination of SAR and optic images in Pott, Amado, Schwalbert, Corassa, and Ciampitti [11] was utilized to increase the accuracy of crop type identification using RF. Their results demonstrated that they were successful in improving the overall accuracy of the classification and preparing more accurate land crop maps.

Previous studies have shown that the accuracy of satellite-based land crop maps relies on (1) the quality of calibration data, (2) characteristics utilized in the process of classification, (3) classification algorithm used for classification, and (4) the complexity of the area. Therefore, to prepare a precise land crop map, these factors must be taken into account. In this regard, selecting the appropriate classification algorithm using satellite images plays a crucial role in the accuracy of the results. Researchers have used different classification methods, which are separated into unsupervised and supervised algorithms or parametric and non-parametric algorithms. Various studies have shown that vector and artificial intelligence algorithms can be more accurate than other algorithms in preparing land crop maps [12,13,14]. However, depending on the type of product and the complexity of the study area, the performance of different algorithms can be different.

Another important factor influencing the accuracy of land crop map is the best possible set of features for the classification process. Different features from different sensors, including optical, radar, and thermal sensors, have different capabilities in identifying different crops. The optimal number of features reduces processing volume, model complexity, and errors, and allows for faster model training, easy understanding, and increases model accuracy and efficiency, thus achieving more accurate results. Therefore, selecting the best features from a large number of features has a significant impact on how well land crops are classified. For this purpose, statistical techniques can be used to minimize additional features and extract effective features. The selection of optimal features should be done in such a way that unnecessary and repetitive features are removed while preserving the original information. For this purpose, feature selection techniques were used. Different models have different strengths and weaknesses that affect classification performance.

Determining and using the appropriate feature selection algorithms with higher performance and the best classification algorithm in land crop mapping according to the conditions of each area is of great importance. The main purpose of this study was to provide a conceptual model to achieve higher accuracy in land crop classification based on a capabilities combination of features derived from different sensors, feature selection algorithms, and different classifier algorithms. In this regard, (1) the variable importance (VI) of different surface features from optical, radar, and thermal satellite images in land crop classification were evaluated, (2) the capability of various feature selection algorithms and classifiers in land crop mapping were compared with each other, and (3) a decision-level fusion method was utilized to create uncertainty maps and increase the classification accuracy of land crops.

## 2. Study Area

**Figure 1:**
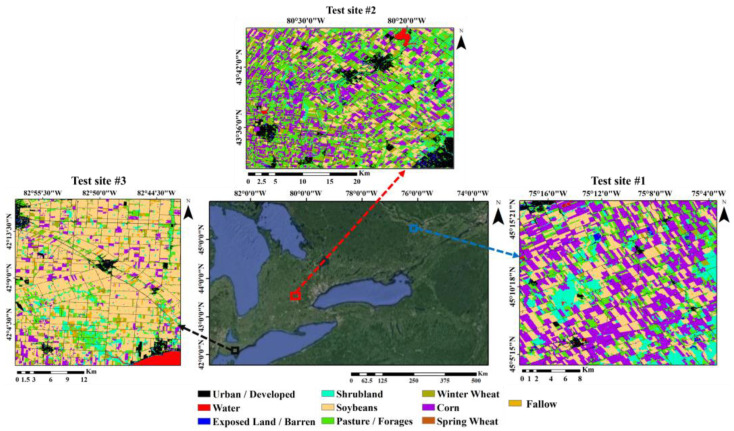
Geographical location of study areas and land crop/cover maps.

Ontario is one of the largest agricultural regions in Canada, but only a small portion of lands in Ontario are arable. Major agricultural products include cereals, soybeans, and corn, with most tobacco farms also located there [15]. Three test sites in Ontario were chosen for this research (Figure 1). The selection of these areas was conducted by considering a variety of agricultural products as well as spatial and geographical diversity in different parts of Ontario.

Test sites 1 (45.1° N, 75.2° W), 2 (43.6° N, 80.4° W), and 3 (42.1° N, 82.8° W) are located in different geographical conditions. The area of these test sites is about 632.7, 682.2, and 915.0 km^2^, respectively. The area under cultivation of dominant agricultural products in these test sites—including corn, soybean, and wheat—is 231.2, 178.0, and 9.4 km^2^ for test site 1; 233.3, 157.7, and 100.9 km^2^ for test site 2; and 62.3, 570.8 and 47.7 km^2^ for test site 3, respectively. Fallow, urban/developed, pasture/forages, exposed land/barren, shrubland, and water are other land covers in these three test sites.

## 3. Data and Methods

### 3.1. Data

Various derived datasets, including Sentinel 1, and 2 and Landsat-8 images time series, were utilized to generate different spectral features maps for classification purposes. The test sites’ land crop reference maps were sourced from https://open.canada.ca/data/en/dataset?q= website (accessed on 16 February 2022) and were used for training and assessing the accuracy of different classification scenarios. Proper spatial, temporal, and spectral resolution, and appropriate temporal coverage of various phenomena over a long period of time, make Sentinel 1 and 2 and Landsat-8 satellite imagery very important and effective in environmental applications.

Cloud cover affects the quality of data recorded by the optical and thermal sensors of Landsat-8 and Sentinel 2 satellites, and thus cloud coverage has been considered in selecting these images. Therefore, Landsat-8 and Sentinel 2 images with less than 10% cloud cover for the test sites were downloaded from https://earthexplorer.usgs.gov/ and https://apps.sentinel-hub.com/eo-browser/ sites (accessed on 20 February 2022), respectively. Sentinel 2 image bands have spatial resolutions of 10, 20, and 60 m, and 12-day temporal resolution. Moreover, the temporal and spatial resolution of the land surface temperature (LST) product obtained from Landsat-8 is 16 days and 30 m, respectively. The spatial and temporal resolution of Sentinel 1 images are 10 m and 12 days, respectively [16]. Sentinel 1 images were derived from the https://apps.sentinel-hub.com website (accessed on 20 February 2022). In several studies, these images were used to map spectral features for classification process [16,17,18,19].

The data recorded by Sentinel 1 does not show the weakness and sensitivity of its optical sensors in the presence of clouds, and the images obtained from these sensors can be used in any weather conditions, even at night. According to one of the goals of this research, which was to examine the effectiveness of several imagery types for classifying land crops, the time-matching of Landsat-8, Sentinel 1, and 2 images was considered to compare the performance of features obtained from different sensors under the same conditions. The initial spatial resolution of the various spectral features obtained from these satellite images was converted to 30 m based on upscaling methods. The dates of the Landsat-8, Sentinel 1, and 2 imagery utilized in this research are presented in Table S1.

The Agriculture and Agri-Food Canada (AAFC) land crop map prepared in 2019 with a spatial resolution of 30 m was utilized to produce the data necessary for calibrating the land crop classification algorithm and to assess the accuracy of the results of the classification of the crops based on various scenarios. This data is produced annually and can be downloaded from https://open.canada.ca/data/en/dataset?q= website (accessed on 16 February 2022).

### 3.2. Method

**Figure 2:**
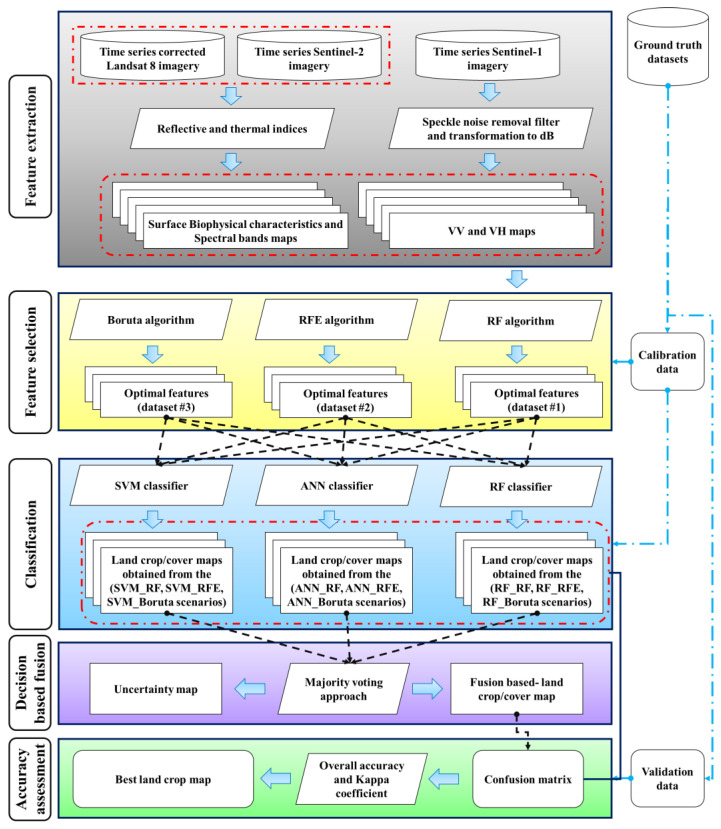
Flowchart of study.

After pre-processing satellite images, the characteristics utilized in the process of classification were extracted based on spectral bands. Then, the optimal features for classification algorithms were determined using training data and various feature selection algorithms including Boruta, recursive feature elimination (RFE) and random forest (RF). After that, various classifiers, including artificial neural network (ANN), support vector machine (SVM), and RF, were implemented for preparing land crop maps according to optimal features and training data. In the following step, to improve the accuracy of the results, maps of land crops derived from several scenarios were integrated. A new land crop map and classification uncertainty map were prepared using a fusion-based voting strategy at the decision level. Finally, different scenarios for land crop mapping were assessed and compared based on accuracy evaluation metrics (Figure 2).

#### 3.2.1. Features Used in Classification

Characteristics considered in the classification procedure included surface biophysical features extracted from Sentinel 2, including impermeability, reflectivity, greenness, and wetness. Surface backtracking properties obtained from Sentinel 1—including co-polarization (VV) and cross-polarization (VH), and land surface temperature (LST) derived from the Landsat-8 surface temperature product—were used as features. Sentinel 2 spectral bands were also considered as features.

Different land crop and cover classes vary in their degree of greenness depending on the season and growth period [20]. The normalized difference vegetation index (NDVI) was used to map the greenness status [21]. In various months of the year, different land crop and cover classes experience variable imperviousness conditions. The IBI index was used to determine imperviousness. The moisture content of agricultural land varies in the period before cultivating, growing, and harvesting (agricultural cycle). Since agricultural crops are cultivated in different times, this feature can be helpful when creating a land crop map. The wetness index derived from the tasseled cap transformation (TCT) was utilized to prepare the map of surface wetness. Different land crop/cover classes can benefit from bright areas with high reflectivity, such as fallows and uncultivated arable fields, as well as dark surfaces and cultivated lands with dense vegetation. The brightness index obtained from the TCT was used to quantify the surface reflectivity. The amount of surface backscattering is determined by the surface topography features and biophysical properties, so it can be used as a parameter affecting the separation of agricultural lands from each other. Various studies have shown that VV and VH are effective parameters on the separation of agricultural lands. Heat is one of the properties that is the result of different surface characteristics. Lands containing different agricultural products before cultivation, cultivation time, different growing periods, and harvest time have different thermal conditions. Therefore, heat can be used as a characteristic in the classification of land crops process. Landsat-8 LST product was used to prepare surface temperature maps.

#### 3.2.2. Feature Selection Process

Optimal features are one of the crucial and influential factors that determine the accuracy of land crop mapping. A lot of characteristics lead to overfitting and increase processing volume and time. On the other hand, considering a small number of features in the classification process leads to not considering effective features comprehensively and reduces the inaccuracy of classification. Therefore, using the optimal number of features will reduce the processing volume, faster model training, reduce model complexity, easy understanding, increase model accuracy and efficiency, reduce errors, and achieve more accurate results. Likewise, selecting effective features from a large number of features from different sensors such as Sentinel 2 spectral bands and surface properties obtained from Sentinel 1, 2, and Landsat-8 in this study can play an important role in improving the results of land crop classification. The RF, RFE, and Boruta algorithms were used as feature selection algorithms, and the effect they had on land crop classification accuracy were compared to one another. Training data for either land crop class was used for implementing these feature selection algorithms. RF is one of the most common algorithms for determining the optimal property in the classification process [22]. In this algorithm, by calculating the VI parameter, the impact of each feature in the classification process was calculated. VI varies between 0 and 100. The details of this algorithm in determining the optimal features are provided in [23]. RFE is a new and accurate way to determine the optimal features before inserting them into classification algorithms. This feature removes inefficient and dependent features. Details of this algorithm for selecting optimal features are provided in [24,25]. In previous studies, this algorithm has been used to determine the optimal features of various applications. However, in this study, for the first time, this algorithm was used to determine the features affecting the separation and classification of land crops. Boruta is one of the prominent methods for preparing optimal features, which is based on RF models. This algorithm determines the importance of a feature by comparing the relationship between real and random probe features [26]. In this study, the features with an importance level of more than 5% in the process of identifying and separating educational data of different land crop classes were selected as optimal features, based on each feature selection algorithm.

#### 3.2.3. Classification Algorithms

Supervised classification algorithms including ANN, SVM, and RF were used to classify the land crop. The results of the previous studies showed that among the various algorithms, the ANN, SVM, and RF algorithms had the highest accuracy in preparing the land cover map [27,28,29,30]. The SVM algorithm uses training samples and their generalization ability to determine the optimal hyperplane and classify and differentiate based on it. This method is suitable for classifying areas with high diversity and high complexity and has the ability to manage time series and multispectral images. The main advantage of this algorithm is the ability to analyze nonlinear relationships, and it uses kernel functions to deal with nonlinear data distribution. The optimal parameters in the implementation of SVM in this study included the radial basis function with a gamma coefficient of 0.143 and the penalty parameter of 100.

The stochastic forest model also uses training data to train a set of trees. After creating the trees and executing the algorithm, the prediction result of each tree is determined separately, and the final output has the highest result of the decision of the trees. The efficiency of RF has been proven for large data, so it is highly efficient in analyzing satellite data. A simple and understandable structure together with a high processing speed are the advantages of this classification method [22]. The number of optimal trees in the RF algorithm was determined to be 150.

ANN handles information similarly to the brain and is inspired by the biological neural system. The neurons that make up this system are numerous and intricately coupled processing units that collaborate to find a solution to a problem. ANNs are trained like humans. During the learning phase, a neural network is configured to carry out certain tasks such as pattern recognition and information categorization. In various studies, this method was employed to classify the land cover [30]. In this study, the multilayer perceptron (MLP) algorithm was used as an ANN algorithm for land crop classification. The optimal parameters for implementing the ANN algorithm were set, including the number of 4 for hidden layers, 400 for training iteration, 0.9 for training threshold contribution, 0.2 for training rate, and 0.9 for training momentum.

About 400 to 500 pixels from the AAFC-created land crop/cover map were chosen to serve as training data for the feature selection and classification algorithms for each land crop/cover class calibration.

#### 3.2.4. Decision-Based Fusion

Each of the feature selections and classifiers algorithms has advantages and disadvantages that affect their performance in land crop classification in different conditions. Therefore, it cannot be concluded that an algorithm is superior in all conditions. A voting strategy can be used to achieve a more accurate result. In this strategy, each class receiving the most votes based on the results of different algorithms was determined as the final fusional class.

Based on this strategy, firstly, land crop classification maps are prepared based on different classification models (in this study, 9 maps were prepared in the first step). Secondly, a linearly vector is formed for each pixel; the number of its elements is equal to the number of different implemented classification models (in this study, each linearly vector for a pixel had 9 elements). In the third step, each element is assigned to a classification model, and the element value is placed with the land crop class code determined for that pixel based on that classification model. Furthermore, the majority operator is executed on each linear vector, and the output value of this operator is introduced as the land crop class code for each pixel based on the proposed strategy. This process is repeated for each pixel separately.

Uncertainty analysis was performed to ensure the accuracy of the generated maps. Uncertainty arises from three sources: the structure of modeling algorithms, model input features, and model parameters [31]. The Equation (1) was used to calculate the uncertainty.

(1) $$\mathrm{Uncertainty}=\frac{\mathrm{Number}\;\mathrm{of}\;\mathrm{classification}\;\mathrm{modes}-\mathrm{majority}\;\mathrm{values}\;\mathrm{frequency}}{\mathrm{Number}\;\mathrm{of}\;\mathrm{classification}\;\mathrm{modes}}\times 100$$

In this scenario, the classification results obtained from the different algorithms are combined. Land crop class is determined for each geographical location based on the agreement of different algorithms. If for a geographical location all the algorithms agree on a type of land crop class, then the uncertainty of determining the type of land crop of that geographical location is very low. However, if each of the algorithms has detected a different land crop class than the other algorithms for a geographical location, then in this geographical location the uncertainty of determining land crop class is very high. Therefore, in this study, based on the fusion-based voting scenario at the decision level, the final land crop map was prepared, and then the land crop classification uncertainty map was determined based on the degree of agreement of different algorithms.

#### 3.2.5. Accuracy Assessment

To assess the performance of various scenarios in preparing the land crop map, omission and commission error metrics were used at the land crop class scale, and overall accuracy was used at the whole areas of test sites scale. A confusion matrix was formed to calculate each of these metrics to assess the accuracy (Table 1). The confusion matrix was created based on the comparison of the land crop/cover maps acquired from various scenarios and the reference land crop/cover map in order to assess the accuracy of the results. The confusion matrix indicates the effectiveness of the algorithms and the outputs of the classification based on the actual information that is now available [32].

The matrix’s rows and columns each include predicted and reference (true) data. The omission and commission errors at the scale of each class and the overall accuracy of the scale of the study area were calculated in this study based on the confusion matrix (Equations (2)–(4)).

(2) $$\mathrm{Commission}\;\mathrm{Error}=\frac{\mathrm{FP}}{\mathrm{FP}+\mathrm{TP}}\times 100$$

(3) $$\mathrm{Omission}\;\mathrm{Error}=\frac{\mathrm{FN}}{\mathrm{FN}+\mathrm{TP}}\times 100$$

(4) $$\mathrm{Overall}\;\mathrm{accuracy}=\frac{\mathrm{TP}+\mathrm{TN}}{\mathrm{TP}+\mathrm{TN}+\mathrm{FP}+\mathrm{FN}}\times 100$$

Test data prepared for each of the land crop classes were used to form the confusion matrix. Test datasets were created using AAFC maps after the calibration process’ pixels had been removed.

## 4. Results

The VI determined for different features using feature selection algorithms varied. According to VI values, among 15 features used from different sensors, the highest and lowest importance was for NDVI and VV, respectively. The mean VI of NDVI and VV for the three test sites were 21.20 and 1.59 for the RF algorithm, 20.33 and 1.01 for the Bruta algorithm, and 21.80 and 1.08% for the RFE algorithm, respectively. Hence, in terms of classifying and separating land crops, NDVI was the most significant factor. In general, based on the mean VI obtained from the three algorithms, NDVI (21.1), IBI (14.3), Wetness (10.9), S2_NIR (8.3), VH (7.2), LST (6.8), S2_Red edge (6.7), and S2_Red (6.3%) were identified the most optimal features for classification, respectively. According to the RF, NDVI (21.2), IBI (15.6), Wetness (14.7), LST (9.3), and VH (6.9%) were the outstanding features in the process of land crop classification. Based on the Boruta algorithm results, NDVI (20.3), S2_NIR (12.5), Wetness (11.4), S2_Red edge (11.3), VH (9.4), S1_Red (9.3), and IBI (8.4%) were selected as the optimal features. NDVI (21.8), IBI (18.8), LST (7.8), and Wetness (6.6%) features were selected as the optimal features used in the land crop classification of test sites based on the RFE algorithm. The results showed that for land crop classification, the VI of the reflective bands of derived features was more than the radar bands-derived features. Moreover, among the surface biophysical properties, greenness, imperviousness, wetness, and heat have been selected as optimal features.

**Figure 3:**
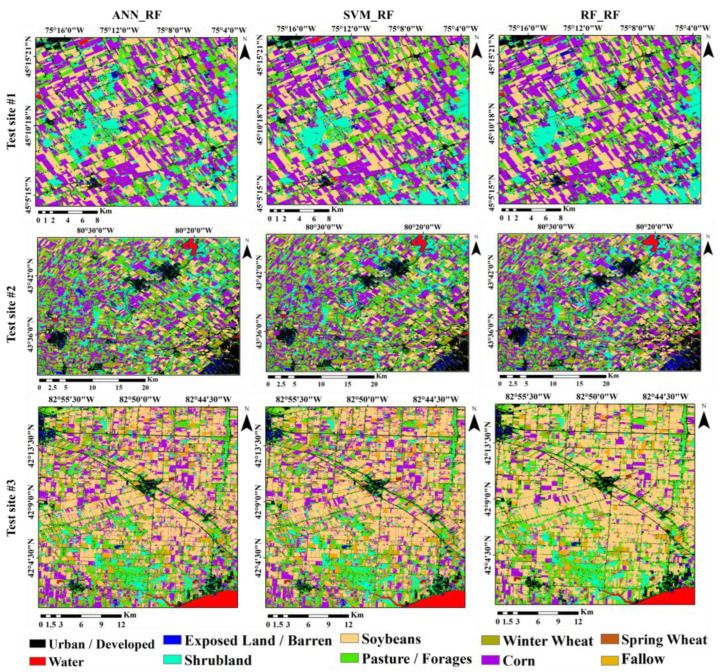
Samples of the land crop/cover maps derived from various scenarios for test site 1, 2, and 3.

Land crop classification maps from nine different scenarios were prepared for the test sites. The results of the three ANN_FR, SVM_RF, and RF_RF scenarios are presented as examples in Figure 3 for the three test sites. Visual study of the maps showed that the results of the scenarios for test sites were different. The noise amount on the RF_RF scenario’s map was less than the other scenarios, and the border areas between the farms were better and clearer than the other scenarios. In addition to the differences in the spatial distribution of land crop classes, each land crop’s area was variable according to the various scenarios. Test site 1 had less in-farm noise than test sites 2 and 3 for each classification scenario.

**Figure 4:**
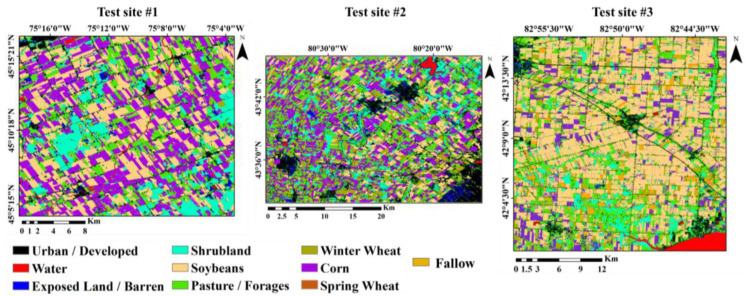
Final land crop/cover maps for the test sites 1, 2, and 3 based on voting scenario.

For more accurate results, the maps from the nine alternative scenarios were integrated based on the voting algorithm (Figure 4). A visual study of voting results showed that the weaknesses of a single-scenario map, including noise, were significantly reduced in voting maps, and the results of voting were more realistic. A comparison of the voting maps with the Google Earth images confirmed this claim. According to the Google Earth images, the resulting maps correspond to the actual boundaries and the type of farm cultivation. The river network, roads, and built-up lands among the farms identified in voting maps were more in line with the Google Earth images than the other scenarios.

**Figure 5:**
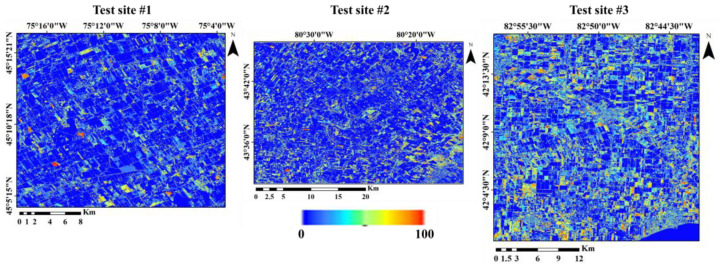
Uncertainty of land crop/cover map obtained from voting strategy for test sites 1, 2, and 3. It is ranged between 0 and 100. The blue color is related to 0 and red color is related to 100.

The uncertainty of the results obtained from the land crop classification was determined using the agreement principle (Figure 5). The lower the agreement between the different scenarios in determining the class of an area, the greater the uncertainty (orange areas), and the higher degree of agreement indicates less uncertainty at the pixel scale (blue areas). The average uncertainty in test sites 1, 2, and 3, based on the voting strategy, was 12, 16, and 21%, respectively. The highest and lowest uncertainties were for test sites 3 and 1, respectively; 5, 12 and 15% of the area of test sites 1, 2, and 3 had higher than 60% uncertainty, respectively. A high percentage of high uncertainty areas were related to the border areas between different farms. Therefore, on the uncertainty maps, the patterns were linear and within the farms, and the uncertainty was lower than the border areas. The percentage of uncertainty in agricultural fields was lower than areas with other uses such as urban, barren soil, and pastures.

**Figure 6:**
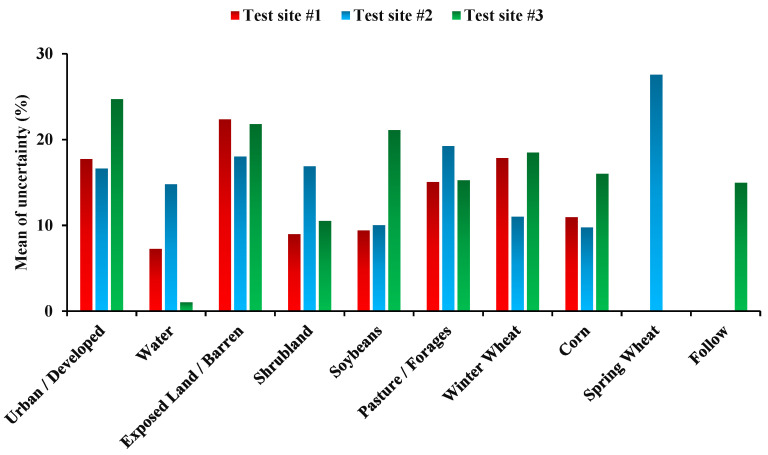
Mean uncertainty of each land crop/cover classes classification for test site 1, 2, and 3.

The mean uncertainty for the classification of different land crops was different (Figure 6). Mean uncertainty for urban/ developed, exposed land/barren, water, shrubland, soybeans, pasture/forages, winter wheat, corn, spring wheat, and follow classes were 19.6, 20.7, 7.7, 12.1, 13.5, 16.5, 15.7, 12.2, 27.5, and 14.9%, respectively. The results showed that spring wheat had the most uncertainty, and water the least, compared to others.

Based on the commission and omission error results for various scenarios, the modeling accuracy of each scenario was evaluated (Table 2). Wheat crop errors had the highest amount compared to other crops. Among different areas, test site 3 had the highest error. The RF_RF scenario with the least error had the best performance in determining the land crop class compared to the other eight scenarios. Based on these results, the amount of error in the voting scenario, which was the result of fusion at the decision level, was less than for single scenarios.

**Figure 7:**
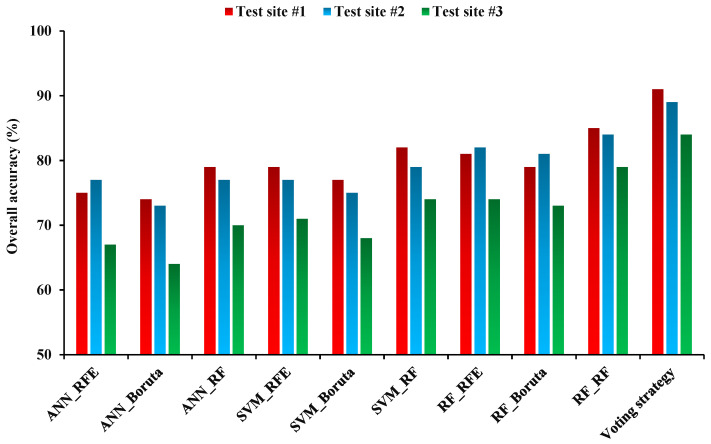
Overall accuracy assessment results for land crop/cover maps in test sites 1, 2, and 3.

The overall accuracy calculated, based on the reference data for maps of the land crops derived from several scenarios, varied (Figure 7). The overall accuracy of ANN_Boruta scenario was lower than other scenarios (74, 73, and 64% for test sites 1, 2, and 3, respectively), which indicated the poor performance of this scenario compared to the others. The highest performance of single scenarios was related to RF_RF (85, 84, and 79% for test sites 1, 2, and 3, respectively), which showed that this scenario performed better. Among the feature selection algorithms, RF accuracy was higher than RFE and Boruta. Additionally, the RF classifier proved more effective than SVM and ANN at creating maps of land crops. Test site 1 had a higher accuracy of land crop developed based on various scenarios than test Sites 2 and 3. The overall accuracy of the voting scenario (91%, 89%, and 84% for test sites 1, 2, and 3, respectively) was higher than all single scenarios, which showed that this strategy has the best accuracy and least error for classifying land crops (Figure 7).

## 5. Discussion

Achieving a thorough land crop map is crucial for agricultural management and planning. In recent decades, various methods have been proposed to prepare this map. In this study, it was shown that the data obtained from satellite imagery had a high efficiency in preparing land cover maps. Results from earlier research also demonstrate that the utilization of data obtained from remote sensing, including MODIS, Landsat, ASTER and Sentinel images, were very useful in preparing land crop maps [19,33,34,35]. Satellite images, due to their appropriate temporal and spatial coverage, can have a high ability to determine the type of land crop of an area and its changes over time. However, the precision of satellite-based maps of land crops is dependent on a number of variables, including the classification features and techniques utilized.

This study investigated the significance of several features derived from Sentinel 1, Sentinel 2, and Landsat-8 on the precision of land crop classification. The findings revealed that the VI of different features was different for each test site (Figure 3). Among the various features, NDVI and VV had the highest and lowest VI, respectively. In different studies, the features that were used to classify land crops accurately were contrasted with one another. Chakhar, Hernández-López, Ballesteros, and Moreno [16] also showed that the efficiency of NDVI obtained from Sentinel 2 in separating land crop types was higher than the backtracking properties obtained from Sentinel 1. Furthermore, in this study, for the first time, a number of characteristics such as imperviousness and heat were used to improve the accuracy of land crop maps, and the results showed the appropriate VI of these features in classification.

The efficiency of feature selection algorithms was compared to determine the VI of different features in land crop classification. The VI calculated for different features varied based on RF, RFE, and Boruta algorithms. The results showed that the efficiency of the RF algorithm was higher than the other two feature selection algorithms. The results of a number of previous articles have also shown the high efficiency of RF in determining the VI of different features in different types of classification [36]. On the other hand, the performance of ANN, SVM, and RF as classifiers in land crop classification were compared. RF efficiency was higher than ANN and SVM for all three test sites. Some of previous studies have shown that the efficiency of this algorithm was higher than ANN and SVM. Nevertheless, the number of parameters that must be defined in the classification process in ANN and SVM algorithms was more than RF. The implementation time of the RF algorithm for classification was less than SVM and ANN algorithms. SVM processing time was also shorter than ANN. RF can work well with thematic and classified data as a feature in classification, but SVM and ANN had weaknesses in this field [37]. If the number of input features in the classification was high, ANN performance can be higher than RF and SVM [37].

To increase the classification accuracy of land crops, the fusion-based voting strategy at the decision level was presented with a set of land crop maps derived from several scenarios (binary combinations of the feature selection and classification algorithms) and the results showed that by implementing the voting strategy, land crop map accuracy was significantly improved. In some of previous studies, fusion at the decision-level was used to improve the accuracy of urban land use/cover maps [38,39,40,41]. One of the limitations of the method presented in this study for land crop classification is the high implementation time and processing volume. However, the processing volume is higher than the presented methods in previous studies, so as to improve the classification accuracy of the final land crop map.

## 6. Conclusions

Land crop mapping is of great importance in improving agriculture, the environment, and natural resources. Recently, several strategies have been suggested for land crop mapping. The characteristics utilized in the classification procedure, and the classification algorithms used, affect the accuracy of the land crop map. Therefore, in this research, the VI of different surface features obtained from optical, radar, and thermal satellite images during the creation of the land crop map was evaluated first. Then, the capabilities of various feature selections and classifier algorithms were compared when constructing the land crop map. Finally, a fusion method was used at the decision level to improve the accuracy of land crop mapping and uncertainty mapping. The results showed that the VI determined for different features using feature selection algorithms was different. In the classification of land crops, the reflecting band features had a higher VI than the radar and thermal band characteristics. Moreover, among the surface biophysical properties, greenness, imperviousness, wetness, and heat have been selected as optimal features. Uncertainty in identifying different classes of land crops was varied. Among the feature selection algorithms, RF accuracy was higher than RFE and Boruta. In addition, the efficiency of the RF classifier in creating land crop maps was greater than SVM and ANN classifiers. The RF_RF scenario with the least error had the best performance in determining the land crop class compared to the other eight scenarios. The classification error in the voting scenario was less than for the single scenarios. The results of this study showed that combining the capabilities of features obtained from multi sensors in different spectral ranges, feature selection algorithms, and different classifier improves the accuracy of land crop classification. In future studies, the conceptual model presented in this study can be used to enhance the precision of land crop maps throughout the world at the lowest cost. Moreover, it is suggested that future studies evaluate the effectiveness of the proposed strategy in areas with other agricultural products or even different climatic and environmental conditions. It is suggested to investigate the efficiency of meta-heuristic optimization algorithms in determining the optimal features and enhancing the accuracy of land crop mapping in future studies.

## Figures and Tables

**Table 1:** Confusion matrix schematic design. FP is the false positive, TP is the true positive, FN is the false negative, and TN is the true negative.

		Reference Values	
		Positive (1)	Negative (0)
**Predicted values**	**Positive (1)**	TP	FP
	**Negative (0)**	FN	TN

**Table 2:** Mean of commission and omission errors for each land crop/cover classes at test site 1 (2, 3).

	Soybeans	Corn	Wheat
ANN_RFE	14 (19, 35)	24 (20, 39)	38 (20, 40)
ANN_Boruta	17 (24, 39)	23 (18, 43)	41 (22, 44)
ANN_RF	13 (18, 27)	20 (16, 34)	30 (19, 36)
SVM_RFE	15 (22, 30)	25 (21, 35)	35 (21, 43)
SVM_Boruta	16 (18, 36)	27 (18, 39)	39 (23, 45)
SVM_RF	14 (17, 27)	21 (19, 31)	31 (18, 38)
RF_RFE	12 (17, 28)	17 (15, 32)	32 (17, 35)
RF_Boruta	11 (18, 27)	18 (17, 36)	34 (20, 38)
RF_RF	10 (15, 25)	14 (12, 27)	28 (15, 31)
Voting strategy	8 (12, 19)	11 (10, 22)	24 (12, 26)

## Data Availability

The data used to support the findings of this study are available from the corresponding author upon reasonable request.

## Supplementary Materials

The following supporting information can be downloaded at: https://www.mdpi.com/article/10.3390/s22197428/s1, Table S1: Acquisition times of Landsat-8, Sentinel 1 and 2 images.
