# Supplementary material for: An Innovative Fusion-Based Scenario for Improving Land Crop Mapping Accuracy
Source: Sensors (Basel). 2022 Sep 30;22(19):7428. doi: 10.3390/s22197428 (PMC9571136; doi:10.3390/s22197428)
Supplement: Supplementary file 1 [file sensors-22-07428-s001.zip › sensors-1923959-supplementary.pdf]

sensors

MDPI

Table S1. Acquisition times of Landsat-8, Sentinel 1 and 2 images.

Test sites	Test site #1	Test site #2	Test site #3
**Sentinel 2**	2019/11/30	2019/3/26	2019/12/21
	2019/10/11	2019/4/17	2019/09/22
	2019/09/21	2019/6/14	2019/07/14
	2019/07/13	2019/7/14	2019/05/05
	2019/07/08	2019/8/3	2019/03/26
	2019/06/23	2019/9/19	2019/02/09
	2019/05/06	2019/10/9	-
	-	2019/10/19	-
	**Test site #1**	**Test site #2**	**Test site #3**
**Sentinel 1**	2019/10/10	2019/3/30	2019/10/01
	2019/08/11	2019/04/23	2019/07/09
	2019/07/18	2019/06/22	2019/06/15
	2019/07/06	2019/07/16	2019/05/22
	2019/06/12	2019/08/09	2019/04/16
	2019/05/07	2019/09/14	2019/03/23
	2019/04/24	2019/10/08	2019/02/03
**Landsat-8**	2019/04/13	2019/03/26	2019/04/09
	2019/05/08	2019/06/14	2019/05/27
	2019/06/09	2019/06/30	2019/06/12
	2019/07/27	2019/07/23	2019/07/14
	2019/08/03	2019/08/01	2019/12/12
	2019/10/15	2019/09/18	-
	-	2019/10/20-	-
